# The Yin and Yang of Human Beta-Defensins in Health and Disease

**DOI:** 10.3389/fimmu.2012.00294

**Published:** 2012-10-08

**Authors:** Aaron Weinberg, Ge Jin, Scott Sieg, Thomas S. McCormick

**Affiliations:** ^1^Department of Biological Sciences, Case Western Reserve University School of Dental MedicineCleveland, OH, USA; ^2^Department of Medicine, University Hospitals Case Medical Center, Case Western Reserve UniversityCleveland, OH, USA; ^3^Department of Dermatology, University Hospitals Case Medical Center, Case Western Reserve University School of Dental Medicine, Case Western Reserve UniversityCleveland, OH, USA

**Keywords:** human beta defensins, epithelial cells, carcinoma, monocytes, gingiva

## Abstract

Rapidly evolving research examining the extended role of human beta-defensins (hBDs) in chemoattraction, innate immune-mediated response, and promotion of angiogenesis suggest that the collective effects of hBDs extend well beyond their antimicrobial mechanism(s). Indeed, the numerous basic cellular functions associated with hBDs demonstrate that these peptides have dual impact on health, as they may be advantageous under certain conditions, but potentially detrimental in others. The consequences of these functions are reflected in the overexpression of hBDs in diseases, such as psoriasis, and recently the association of hBDs with pro-tumoral signaling. The mechanisms regulating hBD response in health and disease are still being elucidated. Clearly the spectrum of function now attributed to hBD regulation identifies these molecules as important cellular regulators, whose appropriate expression is critical for proper immune surveillance; i.e., expression of hBDs in proximity to areas of cellular dysregulation may inadvertently exacerbate disease progression. Understanding the mechanism(s) that regulate contextual signaling of hBDs is an important area of concentration in our laboratories. Using a combination of immunologic, biochemical, and molecular biologic approaches, we have identified signaling pathways associated with hBD promotion of immune homeostasis and have begun to dissect the inappropriate role that beta-defensins may assume in disease.

## Antimicrobial Peptides

Antimicrobial peptides (AMPs) are expressed by various human cells, including the epithelial cells that make up the mucosal linings of the body. These ancient compounds are important for the innate defense of a eukaryotic host. They function within a matter of hours, on a broad spectrum of bacteria, fungi, and encapsulated viruses (reviewed in Weinberg et al., 1998; Zasloff, 2002; Hancock et al., 2006). More recently, we and others have identified that AMPs have the ability to work in conjunction with the adaptive immune system by permitting the host to curb, delay, or avoid microbial growth shortly after an infection.

### hBDs as small cationic AMPs of epithelial cell origin

Numerous organisms synthesize peptides which are processed into smaller hydrophobic or amphipathic, bioactive peptides that act as host defense mechanisms with several designations including; magainins, cecropins, melittins, bacteriocidins, and defensins. In animals, three structural defensin subfamilies, designated as alpha, beta, and theta have been characterized, each possessing a distinctive pattern of three canonical disulfide intramolecular bonds. In mammals, defensins were among the first AMPs to be described. While α-defensins are produced by polymorphonuclear leukocytes and intestinal paneth cells, β-defensins are produced primarily by epithelial cells. A computational search strategy identified 28 new human β-defensin genes in five syntenic chromosomal regions. At least 26 of the predicted genes were found to be transcribed. This study focused on finding β-defensin second exons, the genetic region encoding the mature peptide. It is anticipated that a similar approach could be used to discover all first exon coding sequences and the associated regulatory elements that confer cell specificity and responsiveness to inflammatory stimuli and pathogens (Schutte et al., 2002). An additional five novel beta defensin genes were identified using a bioinformatics approach by Rodriguez-Jimenez et al. (2003) that clustered on chromosome 20p13 and that the transcripts of which were found to be highest in the epididymis of the male genital tract. hBD-2 and -3 are generally expressed at low levels in normal physiological conditions and are induced in response to microbial challenge, whereas hBD-1 is expressed at a low level, with little regulation in response to infection or other stimuli. β-defensins display antimicrobial as well as chemoattractant activities based upon the arrangement of disulfide bonds as well as unidentified structural factors that confer full activity (Taylor et al., 2008). β-defensins are mainly expressed in epithelial cells (hBD-1-3 have been shown to be expressed and secreted in the human oral cavity (Ghosh et al., 2007) and form pores in biological membranes (Duclohier, 1994); their 3-D structures have been previously published (Hoover et al., 2000, 2001).

Beta defensins, specific epithelial cell-derived AMPs (see below), “cross-talk” with the adaptive immune system by interacting with specific chemokine and toll-like receptors on myeloid and lymphoid cells, resulting in modulation of immunocompetent cell responses of the host (Yang et al., 1999; Biragyn et al., 2001, 2002; Quinones-Mateu et al., 2003; Feng et al., 2006; Funderburg et al., 2007; Jin et al., 2010; Rohrl et al., 2010). It is theorized therefore, that surveillance through epithelial cell-derived AMPs functions to keep the natural flora of microorganisms in a steady state in different niches such as the skin, the intestines, and the mouth (Yin, as defined for the purpose of this review). However, there is new evidence implicating some of these beneficial molecules as promoters and contributors to neoplasia (Yang as defined for the purpose of this review). Human beta defensin-3 (hBD-3), when overexpressed in epithelial cell-derived solid tumors, promotes selective chemoattraction of myeloid cells to the lesion site and stimulates them to secrete pro-inflammatory cytokines; i.e., contributing to tumor growth (Kawsar et al., 2009; Jin et al., 2010). This review will highlight recent findings, by our group, demonstrating that human beta defensins are not just antimicrobial and immunoregulatory, but, in select pathological states, can contribute to the exacerbation of neoplastic lesions.

## Human Beta Defensins in the Body

### In health and disease

The discoveries that β-defensins originate in mammalian mucosal epithelium (Diamond et al., 1991; Schonwetter et al., 1995; Zhao et al., 1996; Harder et al., 1997; McCray and Bentley, 1997; Boe et al., 1999; O’Neil et al., 1999; Haynes et al., 2000; Garcia et al., 2001; Harder and Schroder, 2002) has led to the hypothesis that these AMPs function to protect the host against microbial pathogenesis at critical confrontational sites. Our group has extended this hypothesis to also encompass the oral epithelium (Krisanaprakornkit et al., 1998, 2000; Weinberg et al., 1998; Dale et al., 2001). The oral epithelia, and cells derived from it, constitutively express human beta defensin-1 (hBD-1) and can be induced to express hBD-2 and -3 (Krisanaprakornkit et al., 1998, 2000; Weinberg et al., 1998; Dunsche et al., 2002). From an immunoregulatory perspective, β-defensins can engage a number of cell surface receptors to promote chemotaxis. These include the ability of hBD-2 to engage the CCR6 receptor on immature dendritic cells (DC) and T cells, and, in a chemokine manner, recruit these cells to the site(s) of interest (Yang et al., 1999). Moreover, we have shown that hBD-3 can down modulate the HIV co-receptor CXCR4, leading to antagonism of cellular activity (Feng et al., 2006). In addition, antigen presenting cells (APCs) undergo maturation in the presence of hBD-3 via toll-like receptors 1, 2 (Funderburg et al., 2007). We and others have also observed that hBD-3 demonstrates high affinity for interaction with CCR2 on myeloid cells (Jin et al., 2010; Rohrl et al., 2010) resulting in chemoattraction in the absence of the natural ligand MCP-1/CCL2 (Jin et al., 2010). Finally, we have seen that hBD-3 can compete with melanocyte stimulating hormone alpha (MSHα), the natural ligand of melanocortin 1 receptor (MC1r) in myeloid cells (unpublished data). The latter observation suggests that hBD-3 may inhibit anti-inflammatory activity promoted by MSHα since this ligand has been shown to induce IL-10 in cells expressing MC1r (Luger et al., 2003).

In the human autoimmune disease psoriasis; epithelial tissue contains high levels of human beta defensins (Harder et al., 1997; Schroder and Harder, 1999; Zasloff, 2002; Sorensen et al., 2003). Indeed, psoriasis skin was the organ used to identify human epithelial production of beta defensin-2 and 3 (Harder et al., 1997, 2001). The recent discoveries that β-defensins originate in mammalian mucosal epithelium, including human (Diamond et al., 1991; Schonwetter et al., 1995; Zhao et al., 1996; Harder et al., 1997, 2001; McCray and Bentley, 1997; Boe et al., 1999; O’Neil et al., 1999; Haynes et al., 2000; Garcia et al., 2001), has led to the hypothesis that these AMPs function to protect the host against microbial pathogenesis at these critical confrontational sites in healthy tissue as well as immuno-compromised or challenged tissue. In addition to antibacterial and antifungal properties, β-defensins also engage the CCR6 receptor on selected immune effector cells, such as immature DC and T cells and evoke a chemokine response, thereby recruiting these cells to the site of interest (Yang et al., 1999). Furthermore, in addition to inducing dendritic cell maturation (Biragyn et al., 2002) through toll-like receptor 4 (TLR4), murine beta defensin-2 (mBD-2)-based vaccines are proposed to elicit potent cell mediated responses and antitumor immunity (Biragyn et al., 2002; see β-Defensins in Cancer). These recent findings indicate that β-defensins are not only antimicrobial, but may also be playing an important role in immunosurveillance by locally helping break peripheral immune tolerance in organs during acute infection/injury (as discussed further in β-Defensins in Cancer).

Because hBD-2 and hBD-3 are found in extremely high levels in psoriatic tissue, and since it appears that defensins can signal through toll-like receptors that are expressed on cutaneous DC and have been demonstrated on regulatory T cells (Caramalho et al., 2003), it would be of great interest to determine if human beta defensins participate in the activation of DC in psoriasis, i.e., through IL-6 upregulation by DC and releasing effector T cells from local Treg cell suppression.

### hBDs in the oral cavity

**Figure 1:**
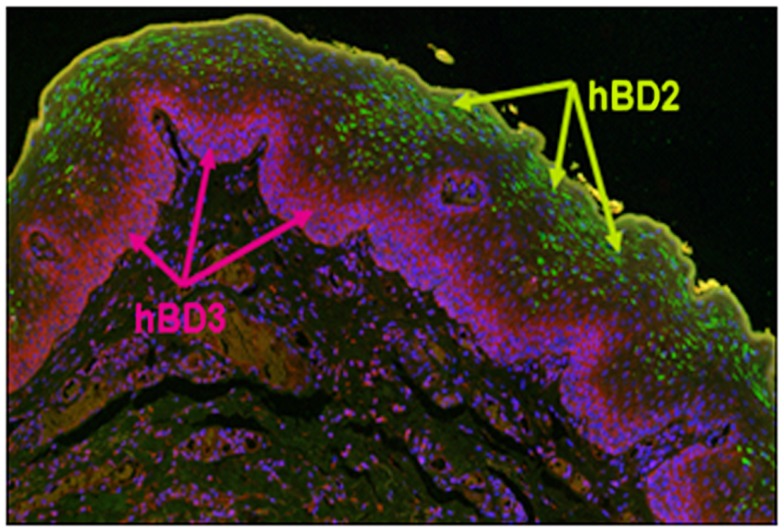
**Distribution of hBD-2 and hBD-3 in normal oral epithelium**. Formalin fixed and parafilm embedded normal human oral tissue was labeled with an anti-hBD-2 specific primary antibody (Santa Cruz, Santa Cruz, CA, USA), and detected with AlexaFluor 488 (Invitrogen, CA, USA; green) conjugated secondary antibody. hBD-3 was labeled with a rabbit anti-hBD-3 (Novus Biologicals Inc., Littleton, CO, USA), specific primary antibody and detected using an AlexaFluor 594 (purple) conjugated donkey anti-rabbit (Invitrogen). Note green fluorescence detection of hBD-2 localized to the stratum spinosum and stratum granulosum, while pink/red fluorescence detects hBD-3 exclusively to the stratum basale. hBD-1, not shown, is localized to the same regions as hBD-2.

In gingival tissue, mRNA for both hBD-1 and -2 is localized in suprabasal stratified epithelium and the peptides are detected in upper epithelial layers consistent with the formation of the stratified epithelial barrier (Dale et al., 2001; Figure 1). Our investigations into the distribution of hBD-3 expression in oral epithelium suggests that while hBD-2 compartmentalizes to the more differentiated stratum granulosum and spinosum, hBD-3 is expressed in the less differentiated stratum basale (Kawsar et al., 2009; Figure 1); further suggesting “cross-talk” capacity between this peptide and resident immunocompetent cells. Most recently, hBD-3 has been shown to be overwhelmingly produced in premalignant epithelial cells in carcinoma *in situ* (CIS) and that this correlates with recruitment and infiltration of monocytes/macrophages exclusively to the lesion site (Jin et al., 2010).

### Epithelial derived AMP-related chemotaxis activity and specificity for receptors

Recent work in the area of epithelial cell-derived AMP activity in recruiting immune cells has led to novel information showing that these ancient molecules act as chemokines in recruiting specific cells from the blood stream. The AMP referred to as LL-37 (named after the first two N-terminal residues and the total number of residues of the mature peptide), the only known cathelicidin like molecule expressed in humans (Zanetti, 2004), attracts immune cells via interaction with the G protein coupled receptor formyl peptide receptor-like 1 (FPRL1; De et al., 2000). We have shown that hBD-3 has no effect on formyl-met-leu-phe receptors, such as FPRL1 (Feng et al., 2006). Instead, hBD-3 interacts with another G protein coupled receptor, CCR2, for chemotaxis of myeloid type cells from the blood stream (Jin et al., 2010; Rohrl et al., 2010). Moreover, while hBD-1 and hBD-2 were found to recruit memory T cells and immature DC via the GPCR CCR6 (Yang et al., 1999), and while hBD-3 can mobilize cells expressing CCR6 *in vitro* (Rohrl et al., 2008), it does not appear to be the case *in vivo*, since hBD-3 exclusively recruits CCR2 expressing myeloid cells at the exclusion of lymphoid cells into oral CIS lesions (Jin et al., 2010).

Collectively, these data suggest that the “promiscuous” nature of hBDs, through interaction with a number of surface associated cellular receptors can orchestrate selective cellular mobility, change phenotypic traits of myeloid cells, and possibly promote a micro environment conducive for inflammation (see Conclusion below).

## β-Defensins as Immune Regulators

### Maturation of iDC and monocytes by hBD-3

The chemotactic responses of human monocytes and iDC to nano-molar concentrations of hBD-3 provides a mechanism for cells to move into inflamed tissues where hBD-3 concentrations may be substantially higher. This raises the question of whether increased exposure to hBD-3 might also influence the maturation and function of these cells as they migrate into inflammatory sites. A key observation from murine studies provided the first indication that defensin molecules could cause activation of APCs via a mechanism dependent on Toll-like receptors (TLRs; Biragyn et al., 2002). TLRs are molecules that recognize conserved microbial structures including, lipopeptides, which are components of bacterial cell walls and are recognized by TLR2, TLR1/2, and TLR1/6 molecules, lipopolysaccharide (LPS), a component of outer bacterial cell membrane that is recognized by TLR4 and unmethylated DNA sequences that are recognized by TLR9. Stimulation of TLRs can lead to signaling mediated by MyD88 and downstream components such as Mitogen Activated Protein Kinases (MAPKS) and Nuclear Factor Kappa-Beta (NF-κB). The activation of APC by a defensin molecule in a TLR-dependent manner suggested that AMPs may not only serve to recruit cells of the adaptive immune system into tissues but may also contribute to the function and maturation of these cells.

In mice, the beta defensin-2 molecule was found to activate APCs via a TLR4-dependent mechanism (Biragyn et al., 2002). Although the relevance of the mouse studies were initially called into question based on the potential for TLR4 contaminants in recombinant defensin reagents (Kopp and Medzhitov, 2002) and due to the lack of a human homolog to mouse defensin-2 (Ganz, 2002), it now seems clear that these initial observations do have relevance to human biology. In particular, human beta defensin-3 has been shown to activate human monocytes in a TLR1/2-dependent manner (Funderburg et al., 2007). Notably, hBD-2, failed to activate cells in a similar manner suggesting that hBD-3 may be uniquely poised to interact with these innate receptors.

Activation of human monocytes by hBD-3 results in several substantial changes in these cells. Notably, hBD-3 induces co-stimulatory molecule expression on human monocytes as well as myeloid dendritic cells (mDC), including the heightened expression of CD80 and CD86 (Funderburg et al., 2007). These co-stimulatory molecules are important ligands for CD28, which is expressed on T cells and plays a critical role in T cell activation. Monocytes activated by hBD-3 also express cytokines associated with inflammation, including IL-8, IL-6, and IL-1β (Funderburg et al., 2011). Importantly, unlike other TLR agonists such as the TLR4 agonists LPS and the TLR1/2 agonist, PAM3CSK4, hBD-3 does not induce IL-10 in these cells (Funderburg et al., 2011). IL-10 is an important regulatory cytokine that suppresses APC function by down-modulating HLA-DR and co-stimulatory molecule expression as well as inflammatory cytokine production by APCs (Fiorentino et al., 1991a,b; Buelens et al., 1995; Chang et al., 1995; Mottonen et al., 1998). Thus, the lack of IL-10 induction by hBD-3 compared to other TLR agonists suggests that hBD-3 may be more prone to inducing a pro-inflammatory microenvironment.

Other investigators have also demonstrated production of both hBD-1 and hBD-2 by DCs albeit at greatly reduced levels of expression. However, in DCs both hBD-1 and hBD-2 were inducible following bacterial exposure, while hBD-3 was only weakly induced. These authors also report that both hBD-2 and hBD-3 up-regulated the expression of IL-8 and GRO in DCs while differential regulation of IL-6 by hBD-2 and MCP-1 by hBD-3 was observed (Yin et al., 2010).

Despite the induction of co-stimulatory molecules and cytokines, not all of hBD-3’s activities seem to be pro-inflammatory. For example, hBD-3 is also capable of modulating innate immunity by neutralization of LPS (Semple et al., 2010). This property is not unique to hBD-3 as other cationic AMPs such as the cathelicidin LL-37, have similar LPS neutralizing activity (Nagaoka et al., 2001; Scott et al., 2011; Suphasiriroj et al., 2012). The neutralization of endotoxin by AMPs is likely to stem from direct binding interactions and the effectiveness of the interaction can be influenced by the source of LPS (Lee et al., 2010). Ultimately, the net effect of hBD-3 on inflammation and bacterial challenge is likely to be complex and will require *in vivo* and *in situ* approaches to fully discern.

### hBD-3 as a mucosal adjuvant

There are relatively sparse vaccine adjuvant approaches that are currently available in clinical practice and few that target T cell immunity and mucosal applications. One recent advance in this area is the development of a TLR4 agonist, monophosphoryl lipid A (MPLA), as an adjuvant (Casella and Mitchell, 2008). This molecule is a derivative of LPS that stimulates cells via TLR4. MPLA provides adequate stimulation for adaptive immune responses but lacks some of the toxicities associated with the parent molecule. The mechanism for MPLA’s success is not fully discerned, although it may be related to a relative skewing of TLR4 signaling in the TRIF-dependent pathway rather than the MyD88-dependent pathway (Mata-Haro et al., 2007). The diminished ability of MPLA to induce certain cytokines in comparison to LPS may account in part, for its better safety profile (Salkowski et al., 1997). The progress made with MPLA has created further enthusiasm for developing TLR agonists as vaccine adjuvants.

Although the full range of hBD-3 activity *in vivo* is likely to be complex and related to the specific tissues and inflammatory conditions in which it is associated, observations made thus far *in vivo* and *in vitro*, have raised the possibility of considering defensin molecules and other AMPs for development as vaccine adjuvants. Immunization studies in mice suggest that hBD-3 and other AMPs can enhance antibody responses to protein antigens *in vivo* (Tani et al., 2000; Brogden et al., 2003; Kohlgraf et al., 2010a,b). hBD-3, for example, boosted IgG responses in mice immunized with intranasal administration of recombinant hemagglutinin B or fimbriae A proteins that are products of *Porphyromonas gingivalis* (Kohlgraf et al., 2010a). hBD-2 mediated similar activity when co-administered intranasally with ovalbumin (OVA) protein (Brogden et al., 2003). Interestingly, murine defensin-2, the TLR4 agonist, was recently used in a tumor vaccination study where the gene for mBD-2 was inserted into B16 melanoma cells and these cells were used for immunization (Mei et al., 2012). Immunization with mBD-2-expressing cells provided protection in mice subsequently challenged with tumor cells and this was dependent on both CD4^+^ and CD8^+^ T cells. NK cell activation was also implicated as a mechanism of protection, suggesting that innate immune mechanisms could also be targeted by this approach. The *in vitro* studies with human cells demonstrate the capacity of hBD-3 to chemoattract APC in a CCR2-dependent manner (Jin et al., 2010; Rohrl et al., 2010) and to further activate these cells in a TLR1/2-dependent manner (Funderburg et al., 2007). Importantly, the identification of these receptor interactions provides a defined mechanism by which hBD-3 can link innate and adaptive defenses.

The involvement of TLR1/2 stimulation in hBD-3 activity may provide some indication of this molecule’s potential adjuvant activity. Although less studied than a number of other TLR pathways for vaccine adjuvant applications, the TLR1/2 signaling pathway was recently shown to be important for immune responses to outer-surface lipoprotein (OspA) in a vaccination strategy for *Borrelia burgdorferi* as mice lacking TLR1 and TLR2 display poor responses to immunization (Alexopoulou et al., 2002). Similarly, TLR2 has been implicated as a critical factor in CD4^+^ T cell responses to the outer membrane protein (OmpA) of *Shigella flexneri* (Pore et al., 2012). Moreover, vaccine adjuvant activity of LT-IIa-B (Rodriguez-Jimenez et al., 2003), derived from enterotoxin, and Chlamydia major OmpA have both been linked to TLR2 activation (Cheng et al., 2011; Lee et al., 2011). Not all studies have shown enhanced adaptive immune responses with TLR2 agonists, however, as these molecules sometimes lead to high induction of IL-10 and potential downregulation of immune responses in certain circumstances (Netea et al., 2004). Thus, the TLR1/2 agonist activity of hBD-3, coupled with its apparent inability to induce IL-10 production from APC (Funderburg et al., 2011), may provide an advantage for induction of adaptive immune responses compared to other more conventional TLR2 stimulants.

## β-Defensins in Cancer

### Expression of human β-defensins in cancers

The discovery of human β-defensins has led to the recognition of the defense mechanism of mucosal surfaces to protect the host from potentially pathogenic organisms. In addition to their antimicrobial activity, the expression of β-defensins in various tissues and cell types has been linked with chemotaxis and innate immune signaling, adaptive immunity, wound healing, and carcinogenesis. Abiko et al. (1999) were the first to describe the detection of human β-defensin-1 and -2 mRNAs in oral cancer cell lines and in tumor samples in an attempt to correlate bacterial infection with the process of oral carcinogenesis. Further reports have shown that hBD-2 peptide was observed in cancer cells in the cornified region of well differentiated oral squamous cell carcinomas (OSCC) and in the hyper-keratinized oral epithelium of oral cancer biopsies (Mizukawa et al., 2000; Abiko et al., 2001; Sawaki et al., 2002). In oral mucoepidermoid carcinoma tissues, neutrophils, and cancer cells that constitute the ducts were positively immunostained with anti-α-defensin antibody (human neutrophil peptides, HNPs), whereas epidermoid cells and intermediate cells were intensely stained with the anti-hBD-2 antibody (Mizukawa et al., 2001). In addition to oral cancer, chromophobe renal cell carcinomas and oncocytomas, two common histopathologic subtypes of renal epithelial neoplasms, were immunohistochemically positive for hBD-1 (Young et al., 2003). Recent studies have investigated expression of hBDs in normal and malignant tissues to elicit the role of β-defensins in carcinogenesis. Kawsar et al. (2009) have found that hBDs are spatiotemporally expressed in normal, non-cancerous oral epithelium and precancerous, and malignant lesions of the oral cavity. In normal oral epithelia, co-expression of hBD-1 and -2 is primarily associated with differentiated epithelial cells in the superficial layers, however, hBD-3 is produced predominantly by mitotically active cells in the basal layer, similar to what was reported by Lu et al. (2005) in gingival epithelium (Kawsar et al., 2009). Interestingly, tumor cells in oral CIS lesions exclusively produce hBD-3, correlated with expression of proliferating cell nuclear antigen (PCNA) in these cells (Kawsar et al., 2009). In addition, precancerous cells in moderate dysplastic lesions and mitotically active malignant cells at the edges of terminally differentiated OSCC only produce hBD-3, suggesting that hBD-3 expression may recapitulate the molecular events of malignant transformation of oral epithelial cells. Kesting et al. (2009) investigated hBD-3 expression in paired cancerous and non-cancerous specimens of 45 patients to establish the association of hBD-3 expression and stages of OSCC. The expression of hBD-3 is significantly up-regulated in OSCC samples compared to corresponding healthy oral mucosa and normal mucosal samples. In addition, immunofluorescence stain of hBD-3 is much more intense in OSCC tissues than in healthy mucosa (Kesting et al., 2009). However, no significant dependence of hBD-3 mRNA levels correlating with tumor size, histological differentiation, cervical lymph node status, or stage of the cancers was found (Kesting et al., 2009).

### Regulation of β-defensin expression

Expression of human β-defensins (hBDs) can be induced by transcription factors that are activated following initiation of various signaling pathways. Analyses using cultured oral epithelial cells have shown that hBD-1 is constitutively expressed (Jurevic et al., 2003), whereas hBD-2 and hBD-3 are inducible (Dale et al., 2001; Pazgier et al., 2006; Yang et al., 2007). However, *in vivo* studies using RNA *in situ* hybridization and immunohistochemistry have linked the expression profile of hBDs with the status of cellular differentiation and state of disease, indicating that expression of hBDs is context-dependent (Dale et al., 2001; Kawsar et al., 2009, 2010; Kesting et al., 2009; Jin et al., 2010). Indeed, the cellular proliferation marker, PCNA, is co-localized with hBD-3 expressing cells in the epithelial basal cell layer and in tumors of the oral cavity (Kawsar et al., 2009). In addition, nuclear translocation of β-catenin, a transcription modulator participating in tumorigenesis, has been identified in cells residing only in the CIS lesion, but not in cells of the adjacent normal region (Kawsar et al., 2009). Expression of hBD-3 is induced by epidermal growth factor (EGF) via MAPK kinase MEK1/2, PKC, PI3K, and p38 MAPK signaling cascades, but not the signal transducers and activator of transcription (STATs) proteins in oral epithelial cells (Kawsar et al., 2009). In addition, transactivation of EGF receptor during human skin wound healing stimulates hBD-3 expression in skin keratinocytes (Sorensen et al., 2006). While signaling pathways that regulate expression of hBD-1 still remain unknown, hBD-2 is induced by pro-inflammatory mediators or bacterial products via activation of NF-κB transcription factors and STATs (Boughan et al., 2006; Chung and Dale, 2008). Kawsar et al. (2010) have shown the expression of hBD-2 in microvasculature in the “keratin pearl” region of terminally differentiated OSCC. In addition, endothelial spindle cells in Kaposi’s sarcoma produce hBD-2 (Kawsar et al., 2010). Interestingly, TGFβ-1 induces hBD-2 expression in human umbilical vein endothelial cells, but not in oral epithelial cells, suggesting that the induction of hBD-2 expression by TGFβ is endothelial cell specific (Kawsar et al., 2010). These data indicate that expression of β-defensins can be regulated by multiple signaling pathways and is cell type-dependent.

### β-defensins and tumorigenesis

Studies of β-defensin involvement in tumor development and progression have indicated that β-defensins may play important roles in tumorigenesis. Conejo-Garcia et al. (2004) demonstrated that β-defensins can recruit CD11c^+^ DC precursors through CCR6 into tumorigenic areas where vascular endothelial growth factor-A (VEGF-A) transforms them into endothelial-like cells that engage in vasculogenesis and function as promoters of tumor progression in an ovarian mouse model. In human epithelial ovarian neoplasms, hBD-2 peptide has been detected in most ovarian cancers with the strongest expression in tumor islets, while hBD-3 expression has been identified in all cancer specimens analyzed (45/45; Conejo-Garcia et al., 2004). Interestingly, populations of CD11c^+^ leukocytes isolated from freshly dispersed human ovarian cancer express endothelial as well as DC markers, indicating that tumor cell-derived β-defensin molecules may contribute to the recruitment and, in the presence of VEGF-A, endothelial cell-like differentiation of CD11c^+^ DCs in human ovarian cancer (Conejo-Garcia et al., 2004). Nevertheless, numerous tumor types have been shown to have altered receptor expression that enables the tumors to respond to many growth factors, hormones, and cytokines in an autocrine fashion that have the capacity to promote tumor growth to the detriment of the host. Therefore, we should not be surprised that tumors may also respond to self-produced defensins in a similar manner.

Work by Pantelis et al. demonstrated a decrease in hBD-1 gene expression in pleomorphic adenomas compared to healthy and chronic inflamed salivary gland tissue. They suggest that reduced hBD-1 gene expression and altered hBD-1 protein localization from the cytoplasm to the nucleus might play a potential role in tumor development (Pantelis et al., 2009). Indeed, Wenghoefer et al. (2008) have also previously shown that hBD-1 seems to be shifted from the cytoplasm to the nucleus of malignant salivary gland tumors and these authors hypothesize that the nuclear shift of hBD-1 might be associated with malignancy.

Kawsar et al. (2009) have shown that oral tumor cell-derived hBD-3 is associated with accumulation of CD68^+^ macrophages, but not CD3^+^ lymphocytes, in the tumor site, suggesting hBD-3 involvement in recruitment of tumor-associated macrophages (TAMs). To further elucidate the role of tumor cell-produced hBD-3 in oral tumorigenesis, Jin et al. (2010) have shown that tumor cells in oral CIS lesions produce hBD-3, but not macrophage chemotactic protein-1 (MCP-1/CCL2), a known chemokine for macrophage migration, and that hBD-3 chemoattracts monocytes/macrophages. Pretreatment of human monocytes with MCP-1, which binds to CCR2, or RS102895, the CCR2-specific inhibitor, inhibits hBD-3-induced migration of monocytes, indicating that hBD-3 plays an important role in recruiting TAMs via CCR2 (Jin et al., 2010; Rohrl et al., 2010). In addition, hBD-3 and MCP-1 fail to chemoattract monocytes from CCR2 knockout mice (Rohrl et al., 2010). TAMs often make up a significant part of infiltrating immune cells in the tumor microenvironment thereby establishing the inflammatory micromilieu, which often belies a poor prognosis for the patient (de Visser et al., 2006; Allavena et al., 2008; Biswas et al., 2008). TAMs have poor antigen presenting capacity, exhibit an immunosuppressive phenotype, and release pro-tumor cytokines such as IL-6 and TNFα, upon activation (Pollard, 2004; Allavena et al., 2008). Stimulation of human macrophages with hBD-3 induces expression of tumor-promoting cytokines and chemokines, including IL-1α, IL-6, IL-8, CCL18, CCL24, and TNF-α. These data indicate that hBD-3 is important in establishing a tumor-associated inflammatory environment by recruiting monocytes from the peripheral blood and stimulating TAMs to produce chemokines and cytokines that support growth and progression of tumors (Jin et al., 2010).

## Conclusion

**Figure 2:**
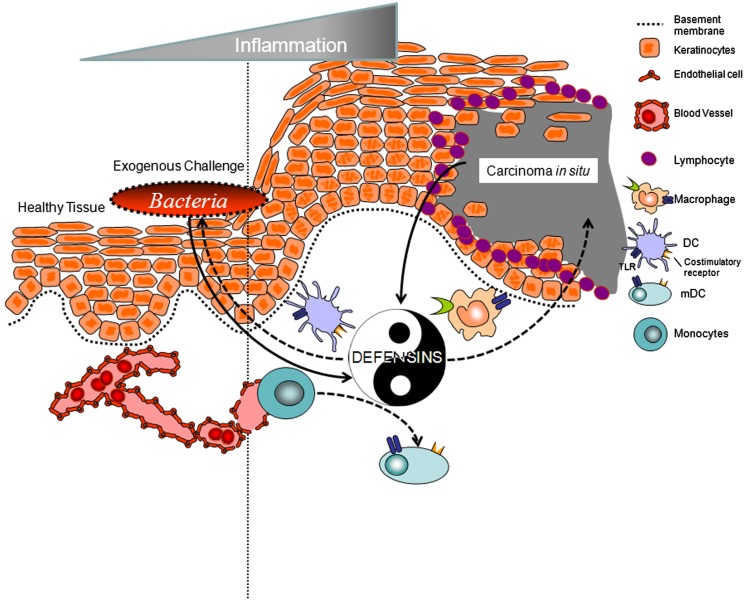
**Yin and Yang of human beta defensins**. Defensins and their homologs are elicited and produced by epithelial cells in response to various stimuli including wounding, bacterial challenge (such as the oral bacteria *Fusobacterium nucleatum*; Gupta et al., 2010), or tumor development (Carcinoma *in situ*, as shown). Defensins modulate the immune-regulatory response to bolster the host response toward challenge. Alternatively defensins may chemoattract immune cells such as tumor-associated macrophages that contribute to tumor-related inflammation and protection of tumors from immune surveillance.

This review brings into question the notion that AMPs are only released by specific host cells to *protect* the host from microbial challenges, be they bacterial, fungal, or viral. AMPs have the capacity to serve as chemokines when secreted in nano-molar (nM) concentrations and as regulators of cell maturity and phenotypic change when present in low micro-molar (μM) concentrations. This is best exemplified by hBD-3 where when present in nM concentrations promotes the recruitment of immature myeloid cells from the blood, via the surface receptor CCR2, to hBD-3 rich sites in the body, such as the stratum basale of the mucosa. Once in the tissue, recruited cells undergo maturation through heterodimerization of TLR 1 and 2 in the presence of low μM levels of hBD-3. Interestingly, in situations where there is overexpression of hBD-3 and little-to-no expression of MCP-1 (CCL2), such as in oral CIS, there is chemoattraction and activation of tumor-associated macrophages that contribute to tumor-related inflammation and protection of tumors from immune surveillance. This is the Yin Yang story of hBD-3 (Figure 2). On the one hand defensins and their homologs may one day be exploited to modulate immune-regulatory strategies as a translational option to bolster the native host response toward, for example, microbial challenge; i.e., novel adjuvants; on the other hand, defensins may be a biomarker for epithelial derived solid tumors and possibly a target in anti-neoplastic intervention. Current and future studies will determine if these two divergent possibilities indeed capture the full range of diversity for these antimicrobial molecules.

## Conflict of Interest Statement

The authors declare that the research was conducted in the absence of any commercial or financial relationships that could be construed as a potential conflict of interest.
